# Astrocyte FABP7 Modulates Seizure Activity-Dependent Protein Expression in Mouse Brain

**DOI:** 10.3390/neuroglia6030033

**Published:** 2025-09-03

**Authors:** Adam P. Berg, Shahroz H. Tariq, Carlos C. Flores, Micah Lefton, Yuji Owada, Christopher J. Davis, Thomas N. Ferraro, Jon M. Jacobs, Marina A. Gritsenko, Yool Lee, Wheaton L. Schroeder, Jason R. Gerstner

**Affiliations:** 1 Elson S. Floyd College of Medicine, Washington State University, Spokane, WA 99202, USA;; 2 Department of Organ Anatomy, Graduate School of Medicine, Tohoku University, Seiryo-cho 2-1, Aobaku, Sendai 980-8575, Japan;; 3 Sleep and Performance Research Center, Washington State University, Spokane, WA 99202, USA; 4 Steve Gleason Institute for Neuroscience, Washington State University, Spokane, WA 99202, USA; 5 Department of Biomedical Sciences, Cooper Medical School of Rowan University, Camden, NJ 08103, USA;; 6 Pacific Northwest National Laboratory, Environmental and Molecular Sciences Division, Richland, WA 99352, USA;; 7 Voiland College of Engineering and Architecture, Washington State University, Pullman, WA 99164, USA;; 8 College of Pharmacy and Pharmaceutical Sciences, Washington State University, Spokane, WA 99202, USA

**Keywords:** neural excitability, proteomics, protein translation, lipid signaling, blbp, glia

## Abstract

**Background/Objectives:**

Patients with epilepsy commonly experience patterns of seizures that change with sleep/wake behavior or diurnal rhythms. The cellular and molecular mechanisms that underlie these patterns in seizure activity are not well understood but may involve non-neuronal cells, such as astrocytes. Our previous studies show the critical importance of one specific astrocyte factor, the brain-type fatty acid binding protein Fabp7, in the regulation of time-of-day-dependent electroshock seizure threshold and neural activity-dependent gene expression in mice. Here, we examined whether Fabp7 influences differential seizure activity-dependent protein expression, by comparing *Fabp7* knockout (KO) to wild-type (WT) mice under control conditions and after reaching the maximal electroshock seizure threshold (MEST).

**Methods:**

We analyzed the proteome in cortical–hippocampal extracts from MEST and SHAM groups of WT and KO mice using mass spectrometry (MS), followed by Gene Ontology (GO) and pathway analyses. GO and pathway analyses of all groups revealed a diverse set of up- and downregulated differentially expressed proteins (DEPs).

**Results:**

We identified 65 significant DEPs in the comparison of KO SHAM versus WT SHAM; 33 proteins were upregulated and 32 were downregulated. We found downregulation in mitochondrial-associated proteins in WT MEST compared to WT SHAM controls, including Slc1a4, Slc25a27, Cox7a2, Cox8a, Micos10, and Atp5mk. Several upregulated DEPs in the KO SHAM versus WT SHAM comparison were associated with the 20S proteasomal subunit, suggesting proteasomal activity is elevated in the absence of Fabp7 expression. We also observed 92 DEPs significantly altered in the KO MEST versus WT MEST, with 49 proteins upregulated and 43 downregulated.

**Conclusions:**

Together, these data suggest that the astrocyte Fabp7 regulation of time-of-day-mediated neural excitability is modulated by multiple cellular mechanisms, which include proteasomal pathways, independent of its role in activity-dependent gene expression.

## 1. Introduction

Epilepsy is a neurologic disorder characterized by recurring seizures together with variable cognitive and behavioral abnormalities. It is a complex disease encompassing multiple seizure types, etiologies, and prognoses [1]. Seizures in isolation can be caused by numerous physiologic insults such as infection, stroke, and trauma. They are broadly defined as periods of abnormal, synchronous excitation of a population of neuronal pathways. This period of excitation can last seconds or minutes and the clinical manifestation depends heavily on the region of brain involved [2]. The pathogenesis of epilepsy involves persistent neuronal structural changes that lead to chronic excitability and impaired synaptic functionality [3]. It is a disease in which neurons have chronically maladapted synaptic excitability thresholds. Seizures in patients with epilepsy can occur either randomly or in a predictable chronologic pattern [4]. Sleep and circadian rhythms are well known to influence seizures [5–7]. Different seizure types occur more frequently either in the day or nighttime depending on the lobe from which the excitability originates [8,9]. The relationship between seizures and time of day suggests that circadian changes within cells of the CNS contribute to seizure vulnerability.

Growing evidence indicates that glial cells play a significant role in the pathogenesis of epilepsy [10]. Astrocytes in particular have several functions related to control of neural excitability [11,12] that, if defective, can contribute to the development of epilepsy. For example, they play vital roles in regulating potassium balance and glutamate trafficking [13,14] and exert control over synapses by surrounding them with perisynaptic astrocytic processes (PAPs), thereby regulating neurotransmitter uptake and gliotransmitter release [15]. Astrocytic impairments in neurotransmitter clearance, such as glutamate, can lead to excessive excitatory signaling, promoting seizure activity [16]. Additionally, alterations in astrocytic gap junctions and their ability to synchronize neuronal networks further facilitate seizure propagation [17], emphasizing the importance of astrocyte–neuron interactions in the development and maintenance of epileptic conditions [18–21].

Our previous studies showed a strong time-of-day effect on electroshock seizure threshold in mice [22], an effect which was significantly reduced in knock-out mice for the core clock gene transcription factor, BMAL1 [23], corroborating additional studies which show changes in excitability and epileptic activity in other circadian gene mutant backgrounds [24,25]. One molecule of interest in this context is brain fatty acid binding protein 7 (Fabp7), which is predominantly found in astrocytes and is involved in lipid signaling cascades that regulate changes in cell growth, morphology, and motility [26,27]. Transcription of Fabp7 follows a diurnal pattern and is dependent on the core clock proteins Rev-Erbα and BMAL1 [28,29]. Fabp7 mRNA is trafficked to PAPs and its concentration increases during times of the day that correlate with elevated behavioral activity [30]. Given that Fabp7 enrichment in PAPs coincides with arousal and regulates cell process outgrowth, it is plausible that Fabp7 is part of a signaling cascade that modulates PAP coverage and alters neural excitability.

Here we used tandem mass tag (TMT) labeling combined with liquid chromatography–tandem mass spectrometry (LC-MS/MS) to characterize differentially expressed proteins (DEPs) in four comparisons among four experimental groups of mice: WT MEST versus WT SHAM, KO MEST versus KO SHAM, KO SHAM versus WT SHAM, and all KO versus all WT. We selected ZT20 for our proteomic analysis based on our previous findings showing that (1) Fabp7 exhibits peak protein expression and perisynaptic localization during the dark phase [30,31], and (2) the most robust time-of-day differences in Fabp7-dependent seizure threshold occur during the dark phase, around ZT20 [22]. This highly sensitive quantitative proteomic analysis (LC-MS/MS) followed by bioinformatic processing is a modern approach for identifying global changes in protein expression. This method of analysis offers significant benefits over the older two-dimensional difference gel electrophoresis (2D-DIGE) method or free quantitation (LFQ) method including enhanced accuracy and reliability of quantitative data analysis, allowing for the identification of proteins across a wide range of molecular weights and abundance levels. It also exhibits excellent sensitivity, specificity, accuracy, and reproducibility [32].

## 2. Materials and Methods

### 2.1. Animals and Seizure Threshold

All animal procedures were carried out in accordance with the National Institutes of Health Guide for the Care and Use of Laboratory Animals and approved by the WSU Institutional Animal Care and Use Committee (IACUC; ASAF# 6509; Last Approved: 27 March 2025).

Male C57BL/6N (WT) and coisogenic background *Fabp7* KO mice (provided by Dr. Yuji Owada) were used for all experiments. Mice (12–16 weeks old) were housed on a 12:12 light–dark cycle and given ad libitum access to food and water.

#### Seizure Tests and Tissue Dissection

*Fabp7* KO and WT mice were tested for seizure threshold at 8 h after lights off (zeitgeber time (ZT) 20), using a single electric shock delivered via ear clip electrodes once per day [22]. SHAM mice received the same handling, including ear clip electrode placement, but did not receive the shock. We used a constant current electroshock unit (model No. 7801, Ugo Basile, Varese, Italy) in which the initial current level was set at 20 mA and increased by 2 mA with each successive daily trial until a maximal seizure, defined by bilateral tonic hind limb extension, was observed. Other parameters of the stimulus were held constant (60 Hz, 0.4 ms pulse width, 0.2 s duration). Upon reaching MEST, mice were euthanized immediately, and brains were harvested from WT-MEST (*n* = 4), Fabp7 KO-MEST (*n* = 4), WT-SHAM (*n* = 4), and Fabp7 KO-SHAM (*n* = 4) mice at ZT20, flash frozen, and kept at −80 °C until processing. Unilateral brain tissue encompassing the hippocampus and cortex (−1 to 3 mm AP and 0 to 2.5 mm ML) was blocked on dry ice, and homogenates were used for subsequent protein extraction, trypsin digestion, and LC-MS/MS.

### 2.2. Protein Extraction, Trypsin Digestion, and LC-MS/MS

All tissues and samples were independently coded, and experimenters were blinded to the samples to reduce the potential for bias. Approximately 20 mg of each of the frozen brain tissues was homogenized in 250 μL of cold lysis buffer (8 M Urea; 50 mM Tris HCl, pH 8.0; 75 mM NaCl; cOmplete protease inhibitor cocktail (Sigma 5892791001, Sigma, St. Louis, MO, USA) via hand-held homogenizer (Kontes^™^ Pellet Pestle^™^ Cordless Motor with disposable pestle) for 30 s. Lysates were incubated for 15 min in the Thermomixer at 4 °C and 600 rpm shaking twice, then were precleared by centrifugation at 20,000× *g* for 5 min at 4 °C. Protein concentrations were determined by BCA assay (ThermoFisher Scientific, Waltham, MA, USA). Protein disulfide bonds were reduced with 5 mM dithiothreitol by incubating lysates for 1 h at 37 °C, and subsequently alkylated with 10 mM iodoacetamide for 45 min at 25 °C in the dark. Samples were diluted 4-fold with 50 mM Tris-HCl, pH 8.0 to obtain a final concentration of 2 M urea, prior to the digesting with Lys-C (Wako) at a 1:50 enzyme-to-substrate ratio. After 2 h of digestion at 25 °C, sequencing grade modified trypsin (Promega, V5117) at a 1:50 enzyme-to-substrate ratio was added to the samples and samples were further incubated at 25 °C for 14 h. The reaction was stopped by acidifying the samples with 100% formic acid (Sigma-Aldrich, St. Louis, MO, USA) to a final concentration of 1% formic acid and centrifuged for 15 min at 1500× *g* to clear digest from precipitation. Tryptic peptides were desalted on a C18 SPE cartridge (Waters tC18 SepPak, WAT036820) and concentrated using a Speed-Vac concentrator. Final peptide concentration was determined via BCA Assay. Peptide samples (100 μg) were labeled with 16-plex tandem mass tags (TMT, ThermoFisher Scientific) according to the manufacturer recommendations. Approximately 1.4 mg of the 16-plex TMT-labeled sample was separated on a reversed phase Agilent Zorbax 300 Extend-C18 column (250 mm × 4.6 mm column containing 3.5 μm particles) using an Agilent 1200 HPLC system. Solvent A was 4.5 mM ammonium formate, pH 10, 2% acetonitrile and solvent B was 4.5 mM ammonium formate, pH 10, 90% acetonitrile. The flow rate was 1 mL/min and the injection volume was 900 μL. TMT-labeled peptides were separated into 96 fractions by high-pH reversed phase chromatography and further concatenated into 12 fractions, as previously described [33,34]. For LC-MS/MS analysis, concatenated fractions were dried and re-suspended in 3% acetonitrile and 0.1% formic acid to a peptide concentration of 0.1 μg/μL. Fractionated peptide samples were subjected to a custom high mass accuracy LC-MS/MS system as previously described [35], where the LC component consisted of automated reversed-phase columns prepared in-house by slurry packing 3-μm Jupiter C18 (Phenomenex, Torrance, CA, USA) into 35 cm × 360 μm o.d. × 75 μm i.d fused silica (Polymicro Technologies Inc., Phoenix, AZ, USA). The MS component consisted of a Q Exactive HF Hybrid Quadrupole-Orbitrap mass spectrometer (Thermo Scientific) outfitted with a custom electrospray ionization interface. Electrospray emitters were custom made using 360 μm o.d. × 20 μm i.d. chemically etched fused silica capillary. All other instrument conditions were set as previously described [35].

### 2.3. Data Analysis

LC-MS/MS raw data were converted into dta files using Bioworks Cluster 3.2 (Thermo Fisher Scientific, Cambridge, MA, USA). The key search parameters used were 20 ppm tolerance for precursor ion masses, +2.5 Da and −1.5 Da window on fragment ion mass tolerances, no limit on missed cleavages, partial tryptic search, no exclusion of contaminants, dynamic oxidation of methionine (15.9949 Da), static IAA alkylation on cysteine (57.0215 Da), and static TMT modification of lysine and N-termini (+144.1021 Da) using the MSGF+ algorithm [36] against the M_musculus UniProt database (3_2023, 21,949 entries). The decoy database searching methodology [37,38] was used to control the false discovery rate at the unique peptide level to <0.01% and subsequent protein level to <0.1% [36]. Quantification was based upon initially summing to the protein level the sample-specific peptide reporter ion intensities captured for each channel across all 12 analytical fractions.

#### Availability of Data

Raw spectral data and analysis information are available via the MassIVE database for public accession at # MSV000097187.

### 2.4. Bioinformatic Analysis

Gene Ontology (GO) and Pathway analyses were performed using Web Gestalt. Each comparison (e.g., WT MEST vs. WT SHAM) was performed individually. The protein input consisted of DEPs from a given pair-wise comparison with *p* < 0.01 from Supplementary Table S1 (Table S1). The reference set was the entire set of proteins from the LC-MS/MS quantification (8656 proteins, Table S1). WebGestalt Basic parameters consisted of Over Representation Analysis in *Mus musculus*. Functional databases included geneontology:Biological Process noRedundant, geneontology: Cellular Process noRedundant, geneontology:Molecular Function noRedundant, pathway:KEGG, pathway:reactome, pathway:WikiPathways and pathway:Panther. Analyte Type was set to “Gene/Protein.” Advanced parameters consisted of Redundancy Removal:weighted set cover, min. no. analytes:5, max. no. analytes:2000, Multiple Test Adjustment:BH, Significance Level:FDR at 0.05, no. categories expected:10, no. clusters:10, no. categories visualized:40, and Color in DAG:continuous. Volcano plots were created using VolcaNoseR [39]. Each pair-wise comparison was analyzed using the input required for the online software: Protein, log2FC, and −log10Pval. Axes for all comparisons were set to “Fold Change threshold” −0.1 > x > 0.1, an arbitrary range allowing for optimal visualization of DEPs. “Significance level” was set to y > 2 since −Log10(0.01) = 2. Lollipop plots were created using RStudio version 2025.05.0+496. For each comparison, the “enrichment_results…” txt file output from Web Gestalt was uploaded to RStudio, which allowed for color-coded visualization of enrichment ratio and number of proteins in each category (labeled “size” on plot legends). Protein–protein interaction networks was completed using the STRING (version 12.0) analysis toolkit [40].

## 3. Results

**Figure 1.**
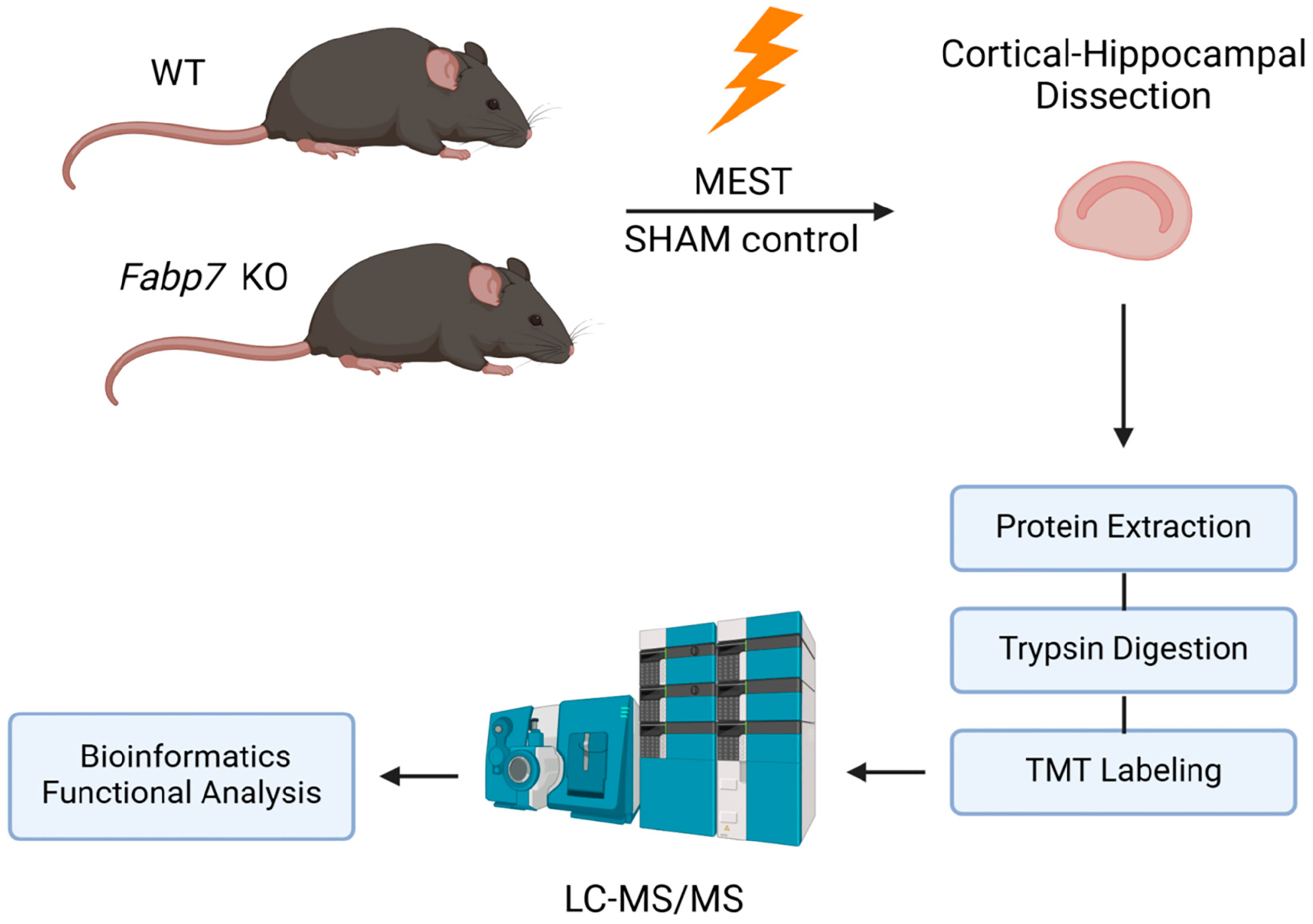
Proteomic workflow schematic. Proteome analysis using LC-MS/MS was performed on cortical–hippocampal samples obtained from Fabp7 KO and WT mice subjected to MEST testing and compared to SHAM controls (*n* = 4/group).

**Figure 2.**
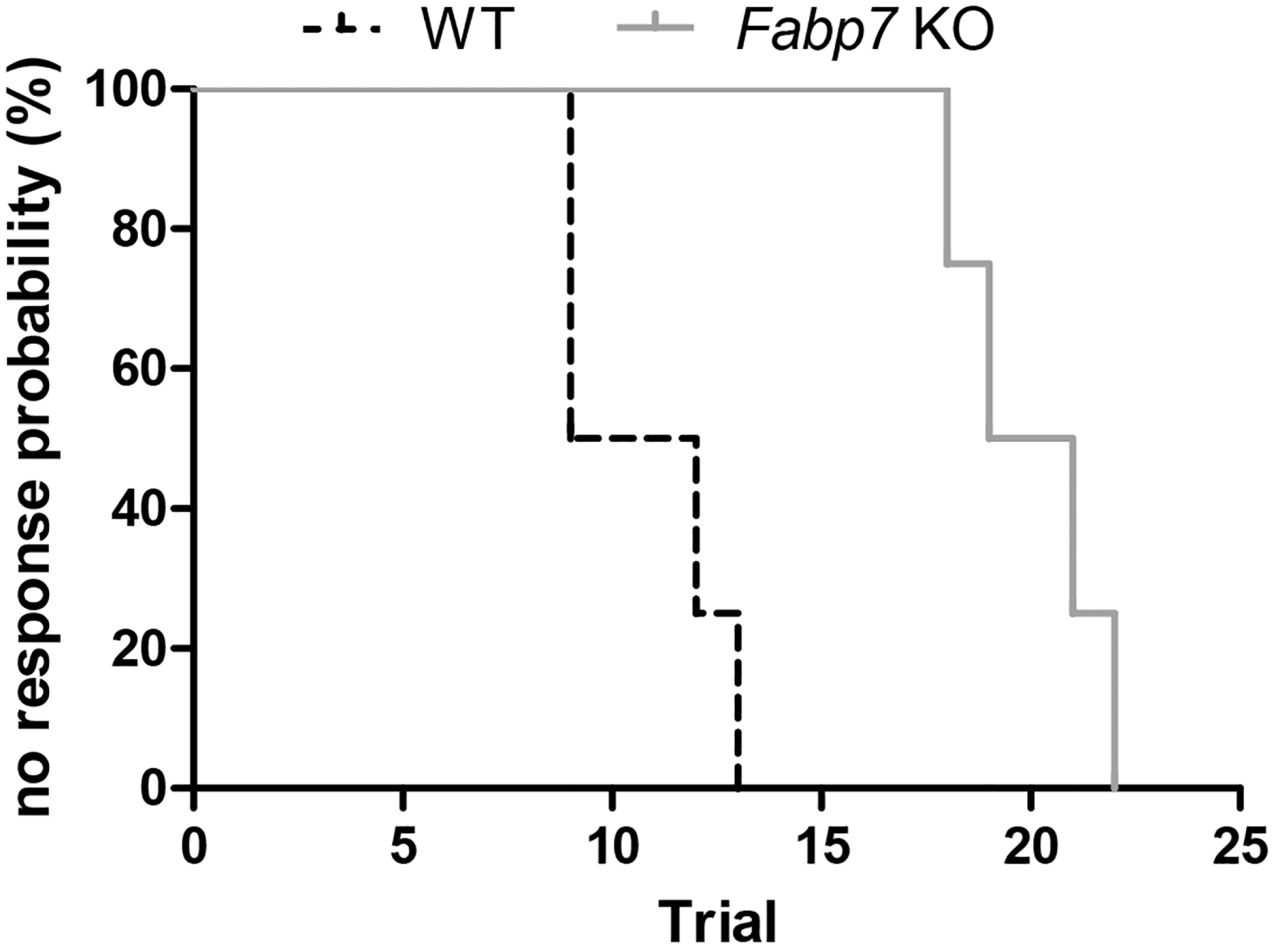
Differences in maximal electroshock seizure threshold (MEST) between *Fabp7* KO versus WT mice used for proteome analysis. Endpoints of electroshock-induced seizures show percent probability of no tonic hindlimb extension (i.e., the response that characterizes the MEST), with higher threshold in *Fabp7* KO mice (solid gray line) compared to WT mice (dashed black line). Median trial number to reach MEST: *Fabp7* KO = 20.0, WT = 10.5; Logrank test: Chi-square 7.5, *p* = 0.0062; *n* = 4 per group. Student’s *t*-test revealed significant differences between *Fabp7* KO mice and WT controls, *p*-value < 0.001; from [22].

**Figure A2.**
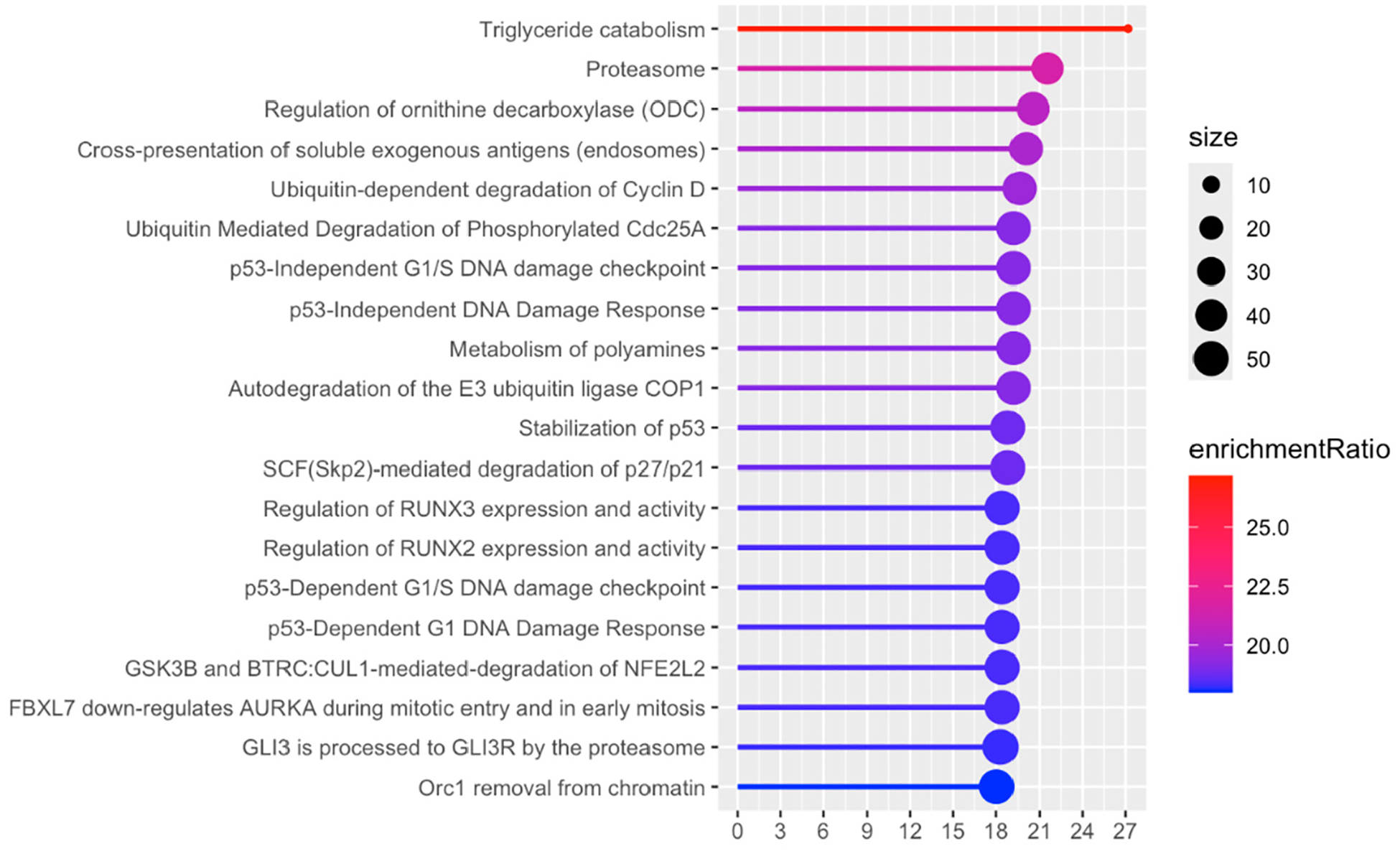
Plot of Pathway/GO terms in KO vs. WT. DEPs restricted to *p*_value < 0.01. “Size” corresponds to total number of proteins in each category.

This study employed a combination of behavioral, proteomic, and bioinformatic approaches to investigate the influence of astrocyte-specific Fabp7 on seizure susceptibility and related protein expression patterns in mice (Figures 1 and 2). Overall, we identified a total number of 8656 proteins in our dataset. Differentially expressed proteins (DEPs) were identified based on statistical thresholds (*p* < 0.01) and analyzed for functional enrichment using WebGestalt to perform Gene Ontology and pathway analyses (Figure 1). Proteome analysis revealed 126 proteins significantly altered in the combined KO versus combined WT comparison (*p* < 0.01); 84 proteins were upregulated and 42 were downregulated (Appendix A). Figure A2 demonstrates the significant pathways of “triglyceride metabolism” and “proteasome.”

### 3.1. Changes in MEST Between WT Versus Fabp7 KO

Here we compared MEST between Fabp7 KO and WT mice, and observed significant increases in Fabp7 KO MEST compared to WT MEST (Figure 2), which were used for subsequent proteomic analysis on isolated cortical–hippocampal brain tissues.

### 3.2. Proteomic Changes

#### 3.2.1. WT MEST vs. WT SHAM

**Figure 3.**
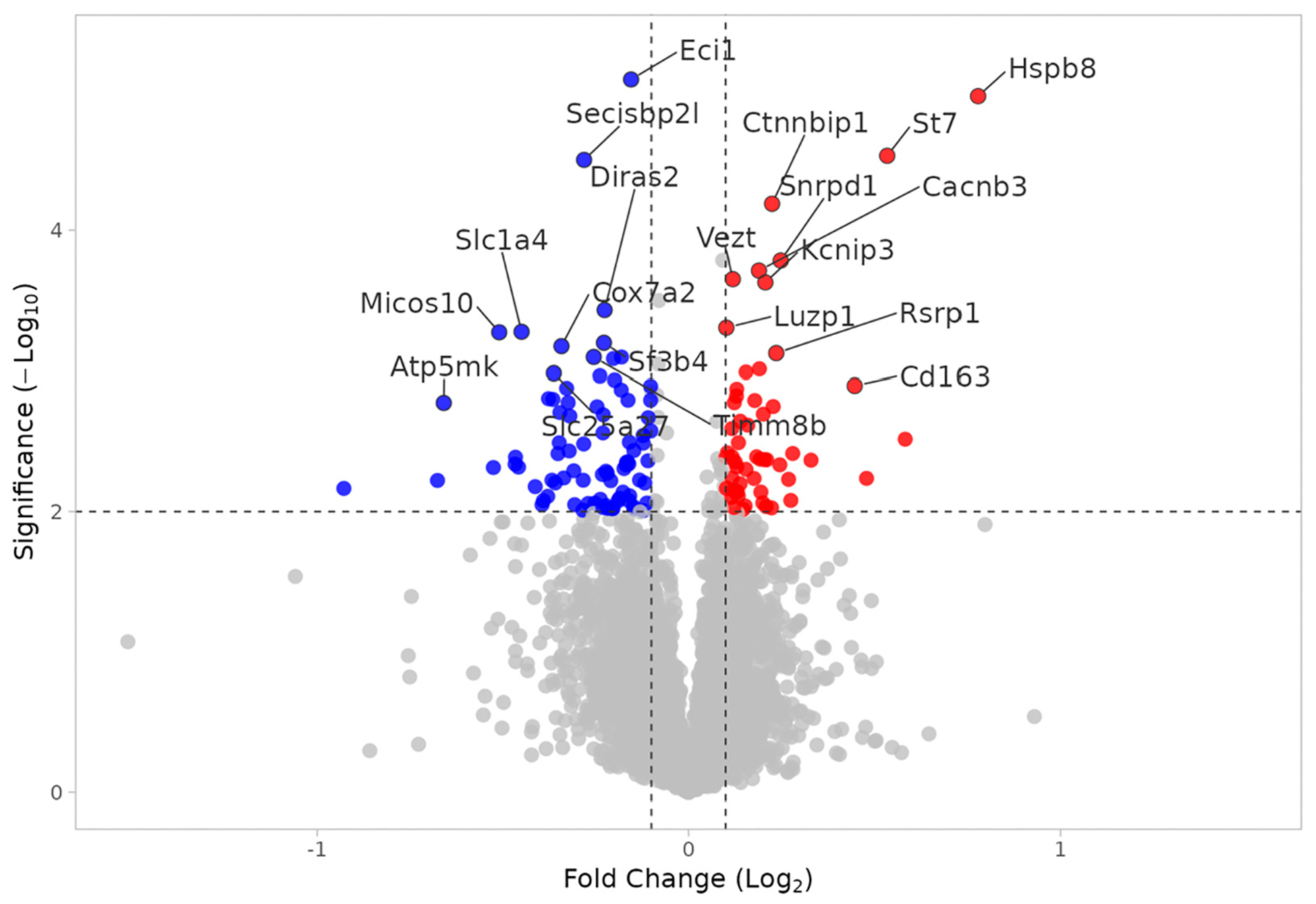
Volcano plot of DEPs in WT MEST vs. WT SHAM. Upregulated (red) and downregulated (blue) DEPs. X axis: Log2(fold change). Y axis: −Log10(*p* value).

**Figure 4.**
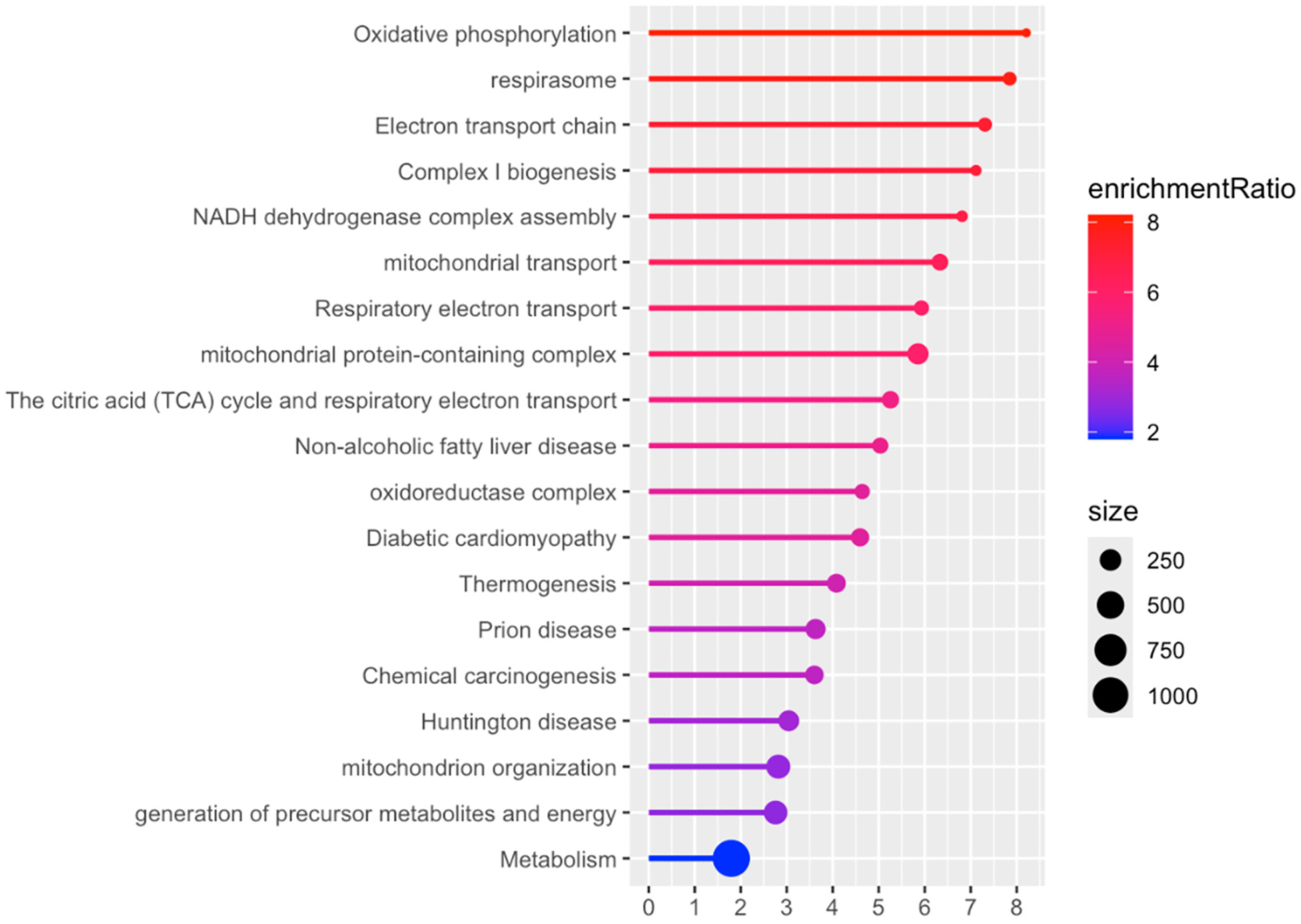
Plot of Pathway/GO terms in WT MEST vs. WT SHAM. DEPs restricted to *p*_value < 0.01. “Size” corresponds to total number of proteins in each category.

Proteome analysis revealed 164 proteins significantly altered in the WT MEST versus WT SHAM comparison (*p* < 0.01); 71 proteins were upregulated and 93 were downregulated. In WT MEST vs. WT SHAM, the difference in protein expression is attributed to the effect of electroshock seizure testing. Figure 3 shows the up- and downregulated proteins in WT animals exposed to electroshock seizures. In WT animals exposed to multiple seizures (i.e., were tested for electroshock thresholds), there is a statistically significant downregulation in mitochondrial-associated proteins compared to SHAM controls, including Slc1a4, Slc25a27, Cox7a2, Cox8a, Micos10, and Atp5mk, which are uniquely implicated in oxidative phosphorylation and the inner mitochondrial membrane. The most statistically significant GO and Pathway terms as shown in Figure 4 support the conclusion that mitochondrial function is profoundly affected by repeated electroshock seizures.

#### 3.2.2. KO MEST vs. KO SHAM

**Figure 5.**
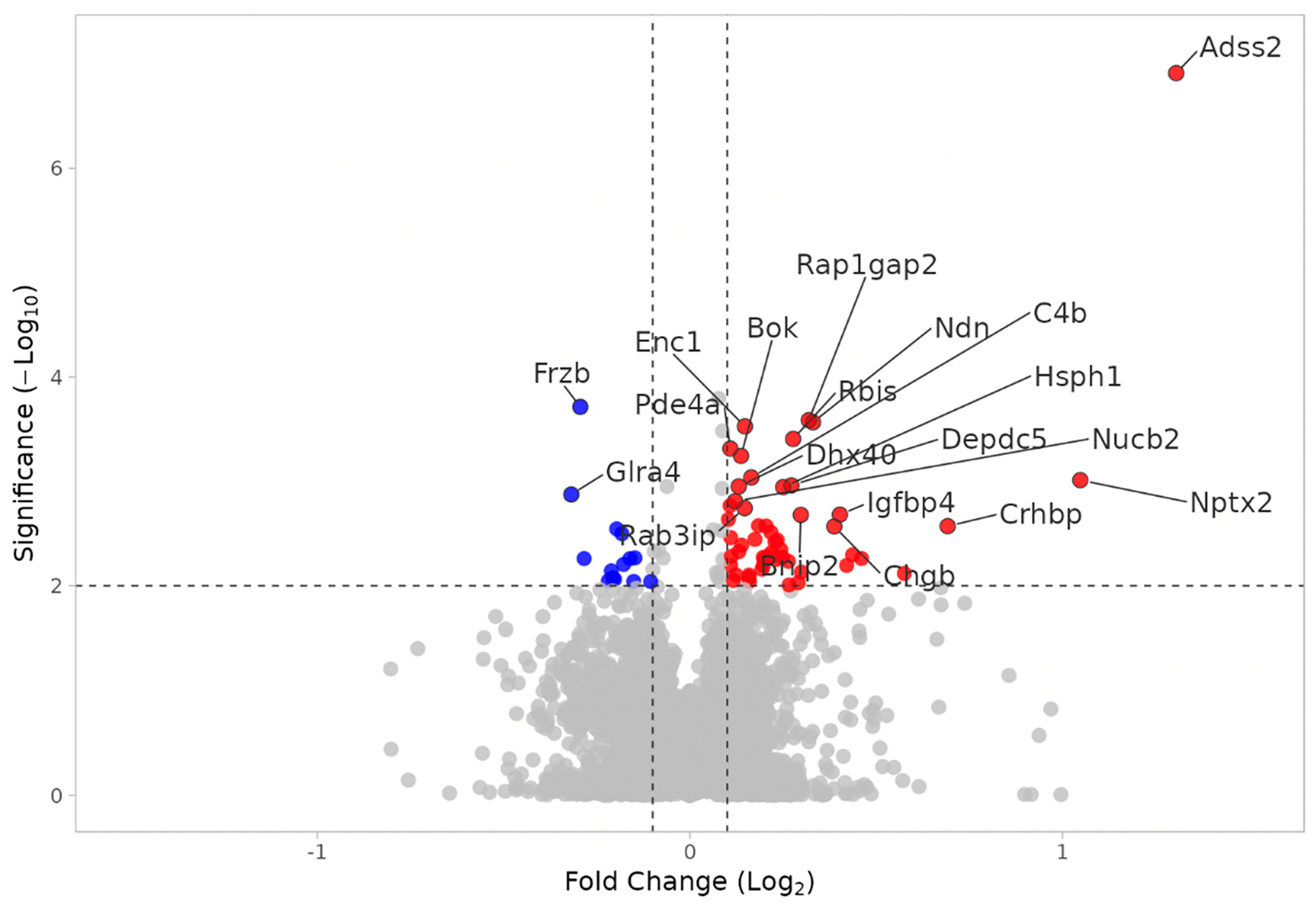
Volcano plot of DEPs in KO MEST vs. KO SHAM. Upregulated (red) and downregulated (blue) DEPs. X axis: Log2(fold change). Y axis: −Log10(*p* value).

**Figure 6.**
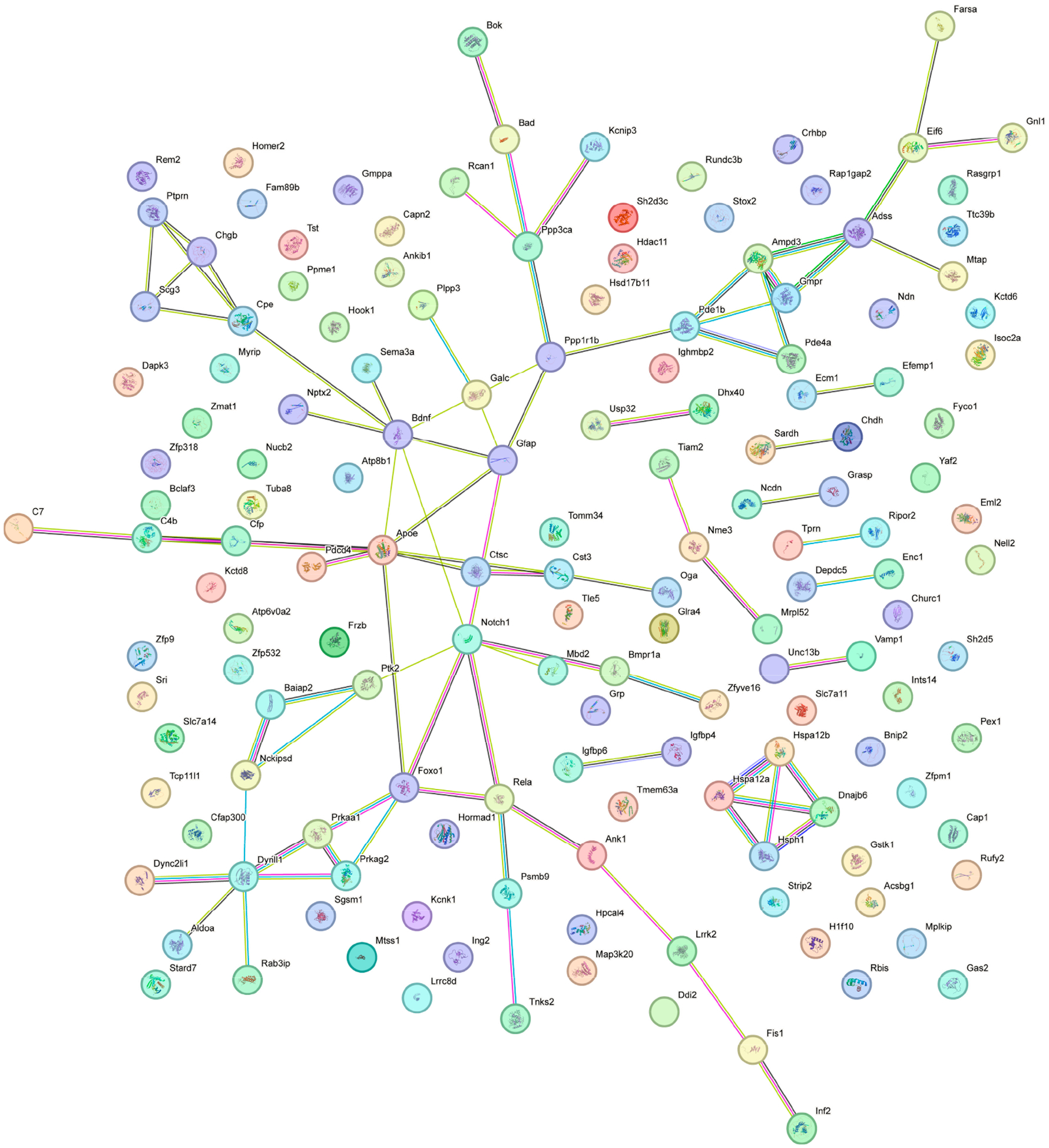
Plot of STRING protein–protein interaction network analysis of DEPs in KO MEST vs. KO SHAM. DEPs restricted to *p*_value < 0.01.

Proteome analysis revealed 153 proteins significantly altered in the KO MEST versus KO SHAM comparison (*p* < 0.01); 105 proteins were upregulated and 48 were downregulated. In KO MEST vs. KO SHAM, DEPs are proteins that are up- or downregulated by electroshock seizure testing in mice that lack Fabp7. The GO term “secretory granule” is the most significant and only output, with an “enrichment ratio” of 4 and “size” of 204. The most significant upregulated DEP is Adss2, as noted in Figure 5. In order to identify functional modules or cores of change, we also examined protein–protein interaction among the KO MEST vs. KO SHAM DEPs (Figure 6), and observed a cluster of “secretory granule” proteins (Scg3, Chgb, and Cpe), as well as heat shock proteins Hspa12b, Hspa12a, Hsph1, and Dnajb6.

#### 3.2.3. KO SHAM vs. WT SHAM

**Figure 7.**
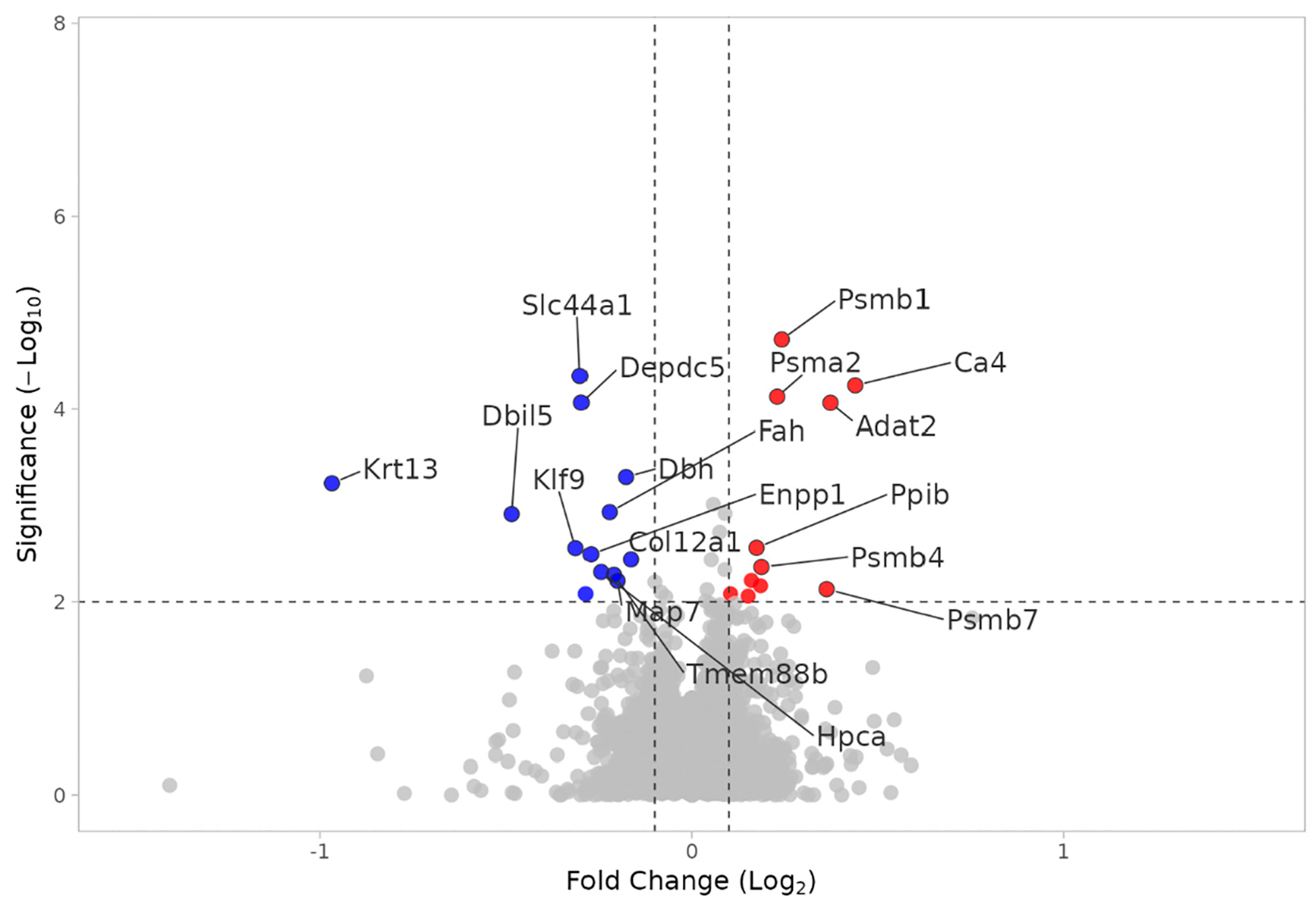
Volcano plot of DEPs in KO SHAM vs. WT SHAM. Upregulated (red) and downregulated (blue) DEPs. X axis: Log2(fold change). Y axis: −Log10(*p* value).

**Figure 8.**
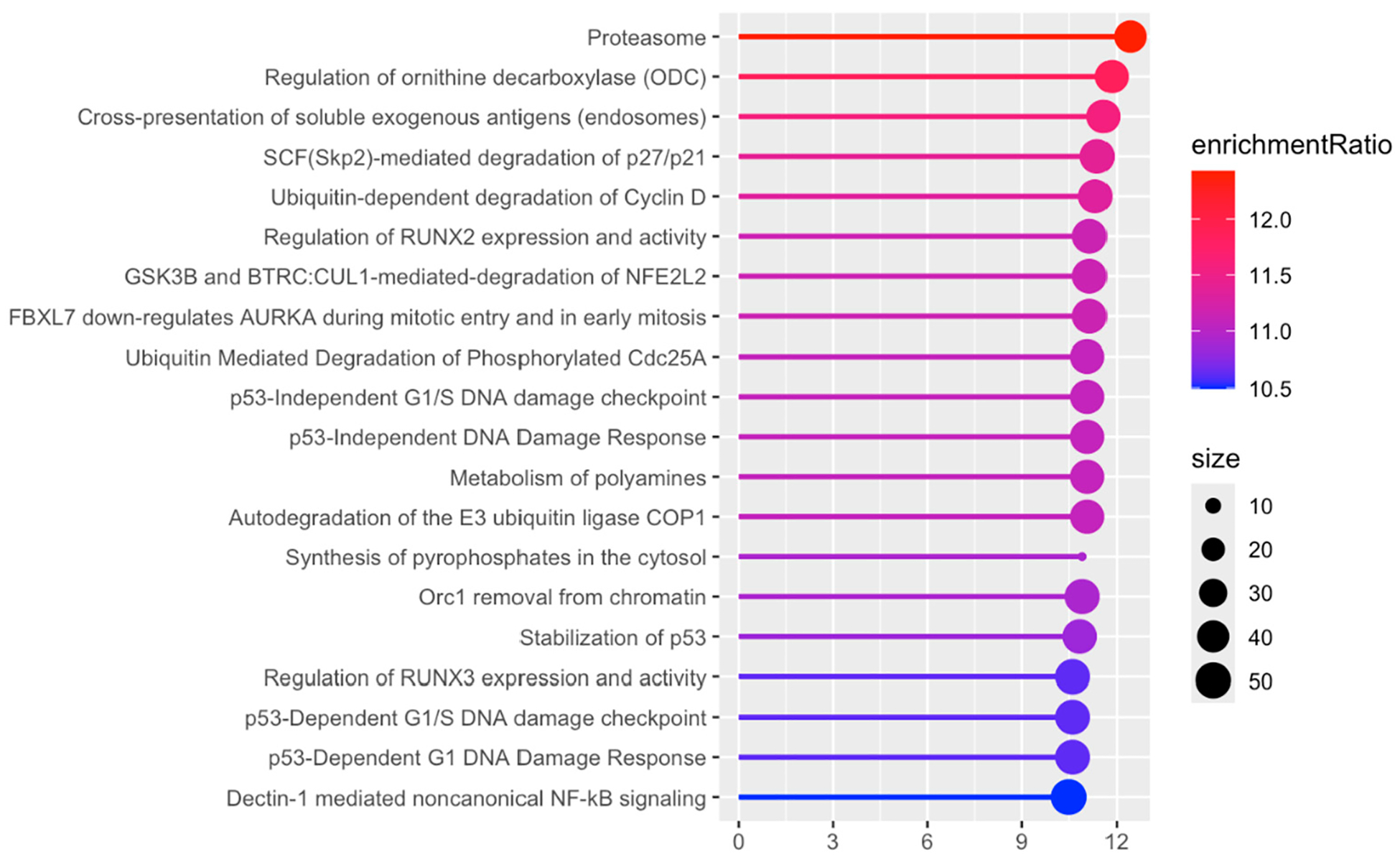
Plot of Pathway/GO terms in KO SHAM vs. WT SHAM. DEPs restricted to *p*_value < 0.01. “Size” corresponds to total number of proteins in each category.

**Figure 9.**
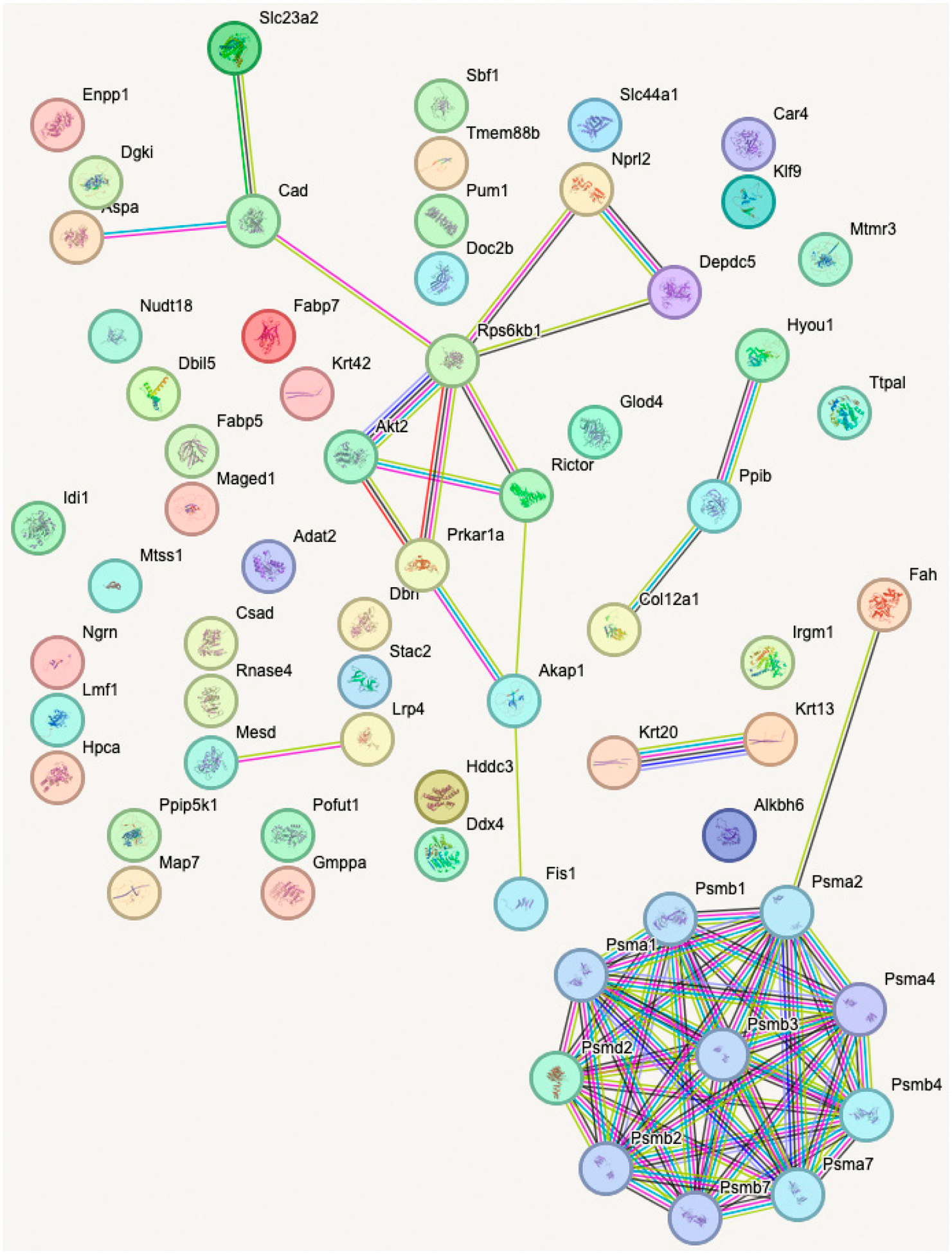
Plot of STRING protein–protein interaction network analysis of DEPs in KO SHAM vs. WT SHAM. DEPs restricted to *p*_value < 0.01.

Proteome analysis revealed 65 proteins significantly altered in the KO SHAM versus WT SHAM comparison (*p* < 0.01); 33 proteins were upregulated and 32 were downregulated. In KO SHAM vs. WT SHAM, the primary focus is the effect of Fabp7 KO. As shown in Figure 7, the Psm family of proteins is upregulated in KO SHAM, indicating that proteasomal function is increased in the absence of Fabp7. The 26S proteasome includes a 20S core subunit, which is composed of two interior beta rings, each enclosed by an external alpha ring [41–43]. The upregulated proteasomal DEPs in Figure 7 are all components of the 20S core subunit: Psmb1, Psmb4, Psmb7, and Psma2. Proteasomal upregulation is supported by the “proteasome” pathway as the most statistically significant pathway result, as shown in Figure 8. In order to identify functional modules or cores of change, we also examined protein–protein interaction among the KO SHAM vs. WT SHAM DEPs, which showed strong interactions among components of the 20S core proteasome, including Psma1, Psma2, Psma4, Psma7, Psmb1, Psmb2, Psmb3, Psmb4, Psmb7, and Psmd2 (Figure 9).

#### 3.2.4. KO MEST vs. WT MEST

Proteome analysis revealed 92 proteins significantly altered in the KO MEST versus WT MEST comparison (*p* < 0.01); 49 proteins were upregulated and 43 were downregulated. Upon Web Gestalt analysis, there were no significant outputs for Pathway or Gene Ontology.

#### 3.2.5. Notable DEPs in KO MEST vs. KO SHAM and WT MEST vs. WT SHAM

**Figure 10.**
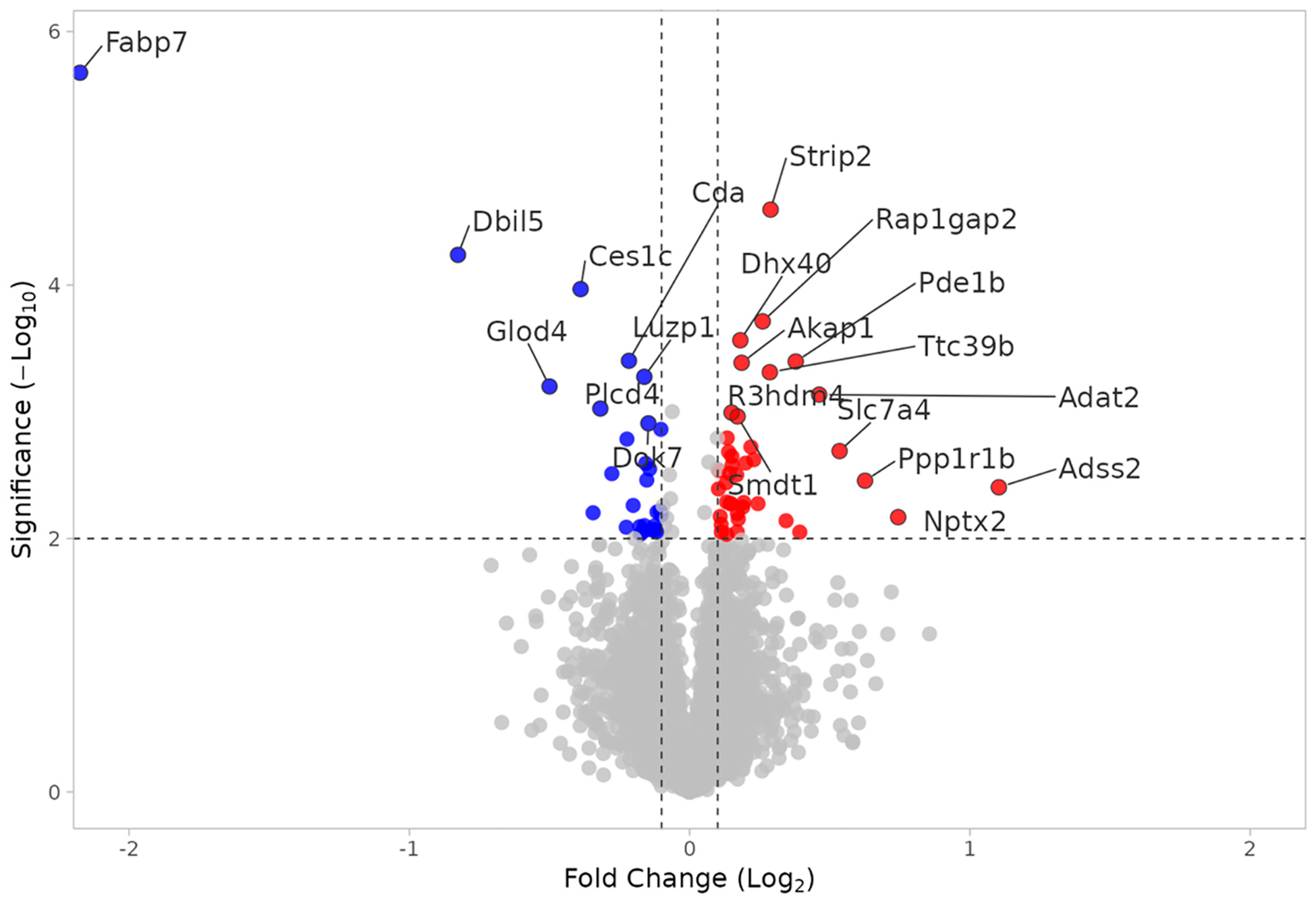
Volcano plot of DEPs in KO MEST vs. WT MEST. Upregulated (red) and downregulated (blue) DEPs. X axis: Log2(fold change). Y axis: −Log10(*p* value).

To better characterize critical proteins regulated by MEST within our study, we compared significant DEPs in KO MEST versus KO SHAM shared with WT MEST versus WT SHAM DEPs not in KO SHAM versus WT SHAM. We identified five DEPs that fit this category, including C4b, Kcnip3, Rap1gap2, Hsph1, and Nptx2, which were all upregulated in the MEST condition regardless of genotype (Table 1). Interestingly, two of these DEPs, Rap1gap2 (0.2605 Log2FC, *p* = 0.002) and Nptx2 (0.7440 Log2FC, *p* = 0.0068), were also found to be significantly upregulated in KO MEST versus WT MEST (Figure 10).

## 4. Discussion and Conclusions

Our previous research demonstrated that Fabp7 plays a role in modulating seizure susceptibility depending on the time of day, showing that Fabp7 KO mice had significantly higher MEST, that is, more resistance to electrically induced seizures, compared to WT mice during the dark phase of a 12 h light/dark cycle [22]. RNA-seq analyses revealed differential expression of many genes, including immediate early genes (IEGs) in cortical–hippocampal tissues from WT mice after reaching MEST versus SHAM procedures during the dark phase, a pattern not observed in Fabp7 KO mice. These findings suggest that astrocyte Fabp7 has dynamic effects on molecular mechanisms tied to heightened neural activity. Here, to better understand the cellular mechanisms underlying the role of Fabp7 in seizure propensity, we performed a quantitative proteomics analysis of brain tissue from Fabp7 KO and WT mice in two conditions, MEST and SHAM.

Among the DEPs in the WT MEST vs. WT SHAM comparison, we identified a downregulation of a group of mitochondria-associated proteins. A previous proteomic analysis of post-mortem hippocampal tissue from patients with epilepsy also identified a downregulation of mitochondria-associated proteins: Slc25a4, Cox6c, Ndufb9, Atp6v1a [44]. Thus, it is possible that downregulation of mitochondrial proteins may be a homeostatic neuronal response to abrogate the oxidative stress caused by electroshock seizures. We observed that Hspb8, a member of the heat shock protein family, is upregulated following MEST in WT mice, but not in KO mice. Many heat shock proteins also serve as chaperones, aiding in protein folding [44]. This may suggest a role for Hspb8 upregulation following seizures as a protective response to oxidative stress through increased protein folding.

We identified the “secretory granule” pathway as the only significant output in KO MEST vs. KO SHAM (Figure 5). Of note, Secretogranin-3 (Scg3), Chromogranin B (Chgb), and Carboxypeptidase E (Cpe) were clustered together in the protein–protein interaction network analysis (Figure 6). Chromogranins/secretogranins, also known as ‘granins,’ are a family of acidic secretory proteins that are found in the secretory granules of a wide variety of cells, including nervous tissue [45,46]. Secretory granules and vesicles assist in exocytosis of proteins and other bioactive materials. Granule types present in astrocytes include densecore vesicles and are known to contain granins [47,48]. Scg3 is involved in the formation and processing of secretory granules, and with Cpe bind to cholesterol-rich secretory granule membranes [49]. Both Scg3 and Cpe are enriched in astrocytes in mice and humans [50,51], however their role in seizure responses is currently unknown. Therefore, the unique discovery of these proteins in the “secretory granule” pathway represents a novel avenue of future research into the role of astrocytes in regulating seizure susceptibility.

**Figure A3.**
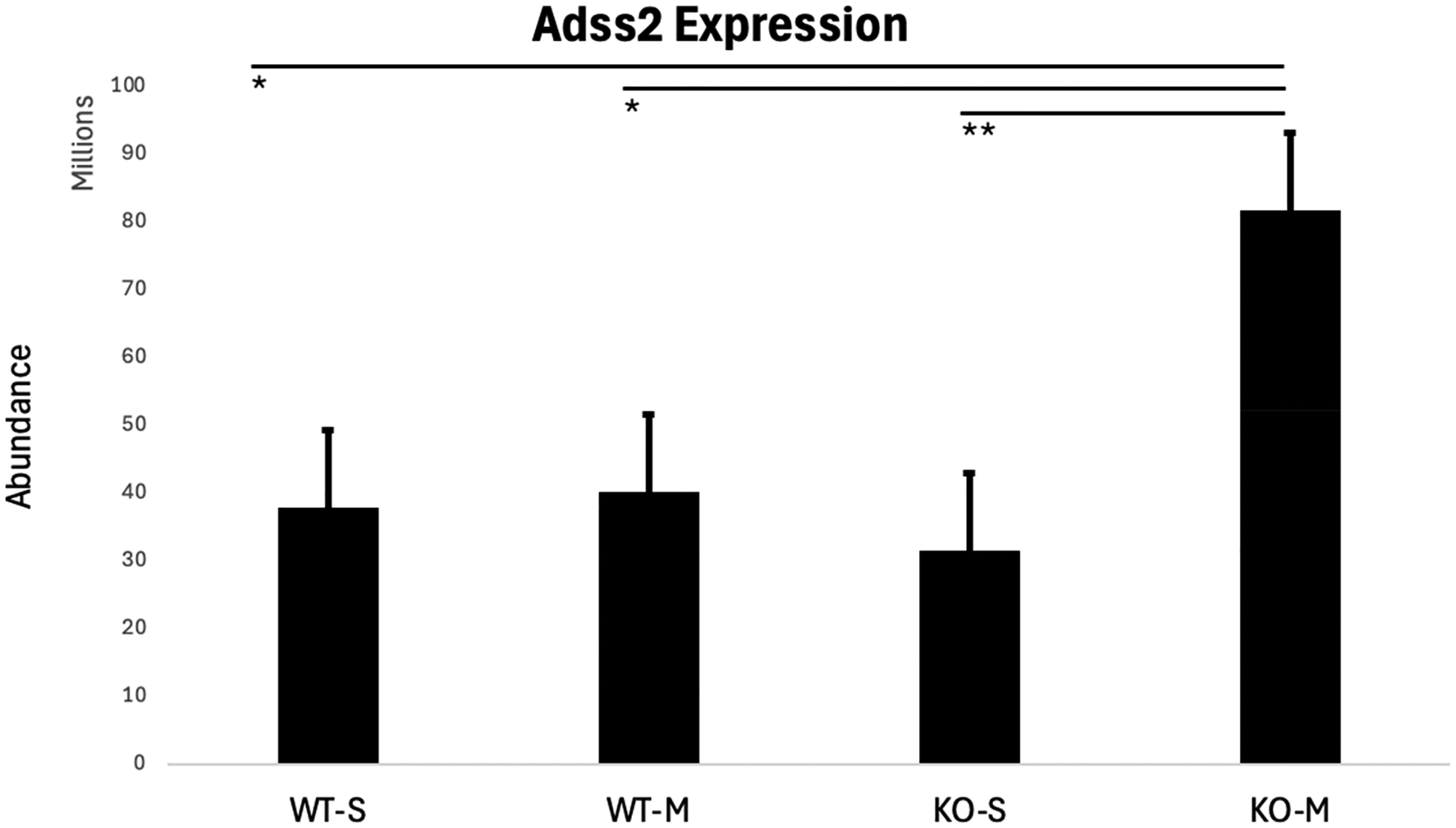
Adss2 protein expression in WT SHAM, WT MEST, KO SHAM, and KO MEST. Adss2 is significantly increased in KO MEST compared to other groups. One-way ANOVA, *p* < 0.001; post hoc Bonferroni WT-S and WT-M, * *p* < 0.01, and KO-S, ** *p* < 0.001 (*n* = 4/group). Effect size *d* = 0.3125 (Cohen’s *d*-value).

There was a significant upregulation of adenylosuccinate synthase 2 (Adss2) in the KO MEST vs. KO SHAM comparison. Vertebrates have two *ADSS* genes, one basic form (*ADSS1*) and one acidic form (*ADSS2*) [52,53]. Adss2 plays an important role in the de novo pathway of purine nucleotide biosynthesis and catalyzes the first committed step in the biosynthesis of AMP from IMP [54,55]. In a previous study, a decrease in Adss2 levels following electroshock seizures in cardiomyocytes was observed [56], suggesting a differential role for AMP synthesis in cells following electrical stimulation. Another study suggests a potential therapeutic role of Adss2 through heterodimerization with Adss1 [57]. Adenosine’s role in neuro-inhibition should also be considered. Research suggests that adenosine is released directly into the extracellular space to potentiate seizure progression, as opposed to an earlier hypothesis that initial exocytosis of ATP followed by breakdown into adenosine was the principal driving factor [58]. In Figure A3 (Appendix B), the TMT mass-spec data show a statistically significant increase in Adss2 protein levels in KO MEST compared to all groups, suggesting a role for astrocyte Fabp7 in repressing neural activity-induced protein expression of Adss2, and implicates glial lipid signaling in purinergic synthesis pathways. While it remains unclear what cell type has Adss2 induced in the KO MEST mice, Adss2 has been shown to be enriched in oligodendrocyte glia [51], suggesting this cell type may be involved in Fabp7-dependent seizure activity in mammalian brains. Adss2 is the most significantly upregulated protein in our analysis, and since Adss2 is involved in AMP synthesis, it may have a role in seizure resistance through intracellular metabolic shifts in adenosine/AMP concentration. This would be important to elucidate further considering the increased seizure threshold in Fabp7 KO mice and the up regulation of both “secretory granule” and Adss2 in the KO MEST vs. KO SHAM comparison. Future studies verifying changes in Adss2 protein expression in KO MEST vs. KO SHAM brain, and the cell-type specificity of differences in expression, will be important for characterizing the relationship between Fabp7 expression and purinergic signaling following changes in neural activity.

Multiple proteins associated with the 20S proteasomal subunit and the “proteasome” pathway were upregulated in the KO SHAM vs. WT SHAM comparison, including Psmb1, Psmb4, Psmb7, and Psma2. High enrichment of the “proteasome” pathway was additionally present in dataset 3.2.4 (all KO vs. all WT). Increases in 20S proteasome DEPs are associated with decreased dendritic spine growth [43]. Membrane-bound 20S subunits are thought to inhibit CaMKIIα activation, decreasing CaMKII-associated recruitment of the 26S proteasome to dendritic spines [43]. The 26S proteasome normally degrades dendritic spine growth inhibitors, facilitating spine growth [59,60]. This observation may help to explain previous observations of anatomical, physiological and behavioral abnormalities in Fabp7 KO mice. Ebrahimi et al. demonstrated that the number and length of dendritic branches and the total area of the dendritic tree all decrease in Fabp7 KO mice compared to WT [61]. Hippocampal NMDA current increases when DHA (an omega-3 polyunsaturated fatty acid that binds to Fabp7 with nM affinity [62]) is introduced to WT neurons, but not Fabp7 KO neurons [63]. In another study, following a challenge injection of MK-801, a potent, non-competitive NMDA receptor antagonist, Fabp7 KO mice exhibited an augmented locomotor response, compared to WT mice [64]. Given the role of the proteasome in degrading spine growth inhibitors, modulating proteasomal activity presents a promising avenue for restoring dendritic structure and function, and implicates the astrocyte Fabp7 in this process. Therapeutically, targeting proteasomal components or their regulatory pathways could alleviate structural and synaptic deficits observed in FABP7-related neurological disorders, potentially improving synaptic activity-related cognitive and/or behavioral outcomes. Although speculative, these insights underscore the importance of further research into proteasome-modulating interventions as strategies to enhance neuroplasticity and general neuronal health in conditions characterized by dendritic and synaptic impairments. While we cannot exclude whether specific DEPs, including proteasomal components, exhibit circadian oscillations independent of Fabp7 from data in the current study, future studies using Fabp7 KO strategies at multiple timepoints over the 24 h day–night cycle will be important for determining if Fabp7 is necessary for these DEPs’ circadian oscillations. Therefore, interrogating the role of the different proteasome subunits in Fabp7 KO compared to WT mice, and their relationship to changes in neuronal excitability in neural–glial interactions, represent important areas of future study.

Despite the 92 DEPs identified in KO MEST versus WT MEST, no pathways reached significance, perhaps reflecting a biological similarity in protein signaling responses to seizure activity between genotypes. In support of this notion, we have identified five upregulated DEPs that are responsive to MEST regardless of genotype: C4b, Kcnip3, Rap1gap2, Hsph1, and Nptx2 (Table 1). Two of these DEPs, Rap1gap2 and Nptx2, were also significantly upregulated in KO MEST versus WT MEST (Figure 10). Rap1gap2, a GTPase-activating protein of Ras-like guanine-nucleotide-binding protein, and Rap1, first thought to play a regulatory role in blood platelet aggregation [65] and secretion by interacting with synaptotagmin-like protein 1 (Slp1) [66], have been identified in neurons of the central nervous system [67,68], and shown to regulate axon outgrowth in olfactory sensory neurons [69]. Nptx2 (Neuronal pentraxin 2, also known as Narp or NP2) belongs to a family of synaptic pentraxins involved in excitatory neurotransmission and acute inflammation [70,71] and has previously been shown to be elevated in epileptic mice [72]. In addition, its inhibition was recently shown to relieve seizures in mice [73]. The upregulation of Nptx2 in KO MEST versus WT MEST may be indicative of the augmented trials of current (mA) application that were required to obtain MEST in the seizure-resistant KO mice compared to WT mice. Future studies determining cell-type expression of Rap1gap2 and Nptx2 following seizure activity will be important for understanding the downstream effects of specific cellular responses in epileptic brain tissue.

Some limitations of the study include the exclusive use of male mice, the examination of MEST versus SHAM at a single timepoint during the dark phase (ZT20) and the low sample size (*n* = 4/group). Future studies expanding the timepoints for more time-of-day resolution and the inclusion of more samples and female mice will greatly help in determining whether the findings might generalize over the diurnal cycle and across sexes. In addition, the method type of seizure induction and/or animal model used may introduce discrepancies in cellular responses in specific molecular pathways, including Fabp7. For example, previous studies using kainate to induce seizure activity show induction of Fabp7 in the hippocampus of rats [74], an effect that was not observed in our previous gene expression study [22] or the current proteomic study, using electroshock.

Our previous studies describe a time-of-day-dependent localization of *Fabp7* mRNA and protein in the PAPs of the tripartite synapse [30], and Fabp7-dependent regulation of sleep–wake behavior across multiple species, including flies, mice, and humans [75]. Increased neural activity during wakefulness, driven by glutamate signaling, leads to fatty acid accumulation and the enhanced production of reactive oxygen species (ROS), which triggers lipid peroxidation [76]. This oxidative process damages neuronal membrane integrity, contributing to cellular dysfunction [77]. We are beginning to appreciate how astrocytes play a crucial protective role by the uptake of fatty acids and glutamate during extended activity/wake periods, particularly at the tripartite synapse, thereby mitigating fatty acid-mediated excitotoxicity [76,78,79]. It is hypothesized that sleep pressure builds concomitantly with increased mitochondrial energy generation in neurons [78,79], coordinated by astrocyte ATP export and conversion into adenosine, a somnogen [80–82]. Sleep facilitates lipid synthesis, membrane repair, and ROS scavenging, processes that influence neuronal excitability and seizure susceptibility [83,84]. High lipid accumulation in astrocytes and dysregulated lipid metabolism are associated with epilepsy and excitotoxicity, contributing to disease progression [85]. Given that astrocytes reduce the number of their fine processes during the dark phase of the circadian cycle, coinciding with circadian differences in NMDA receptor neurophysiology [86], and that Fabp7 KO mice are hyperresponsive to REM sleep rebound following sleep loss during the dark phase [75], Fabp7 may represent a molecular node within astrocytes to coordinate sleep–wake behavior and circadian rhythms, with lipid metabolism following changes in neural activity [79]. While the precise functional role for Fabp7 in regulating these neural processes is not well understood, one compelling mechanism may involve triglyceride metabolism. For example, Fabps retain similar structural and conserved amino acid sequences that include hormone sensitive lipase (HSL) interacting domains, a key enzyme in triglyceride breakdown (lipolysis), which can affect the release of fatty acids from triglycerides for energy utilization and metabolism [87,88]. Future studies determining the functional consequences of Fabp7 loss on excitatory and inhibitory neurotransmission will enhance our understanding of how lipid metabolism and glial-neuronal interactions influence neural circuit balance and susceptibility to neurological disorders such as epilepsy.

## Supplementary Material

Supplemental File

**Supplementary Materials:** The following supporting information can be downloaded at https://www.mdpi.com/article/10.3390/neuroglia6030033/s1.

## Figures and Tables

**Table 1.** Notable DEPs. DEPs in KO MEST versus (v) KO SHAM shared with WT MEST versus WT SHAM DEPs not in KO SHAM versus WT SHAM. Log_2_FC(fold change) and *p*-value/Cohen’s *d*-value (*p*-val/*d*-val).

	KO MEST v KO SHAM		WT MEST v WT SHAM	
DEP	Log_2_FC	*p*-Val/*d*-Val	Log_2_FC	*p*-Val/*d*-Val
C4b	0.1646	0.0009/0.4465	0.1173	0.0041/0.3468
Kcnip3	0.2272	0.0011/0.3029	0.2070	0.0002/0.5864
Rap1gap2	0.3190	0.0003/0.3680	0.1394	0.0023/0.3705
Hsph1	0.2502	0.0011/0.2712	0.2016	0.0020/0.2688
Nptx2	1.048	0.0010/1.9672	0.3300	0.0043/0.4552

**Figure A1.**
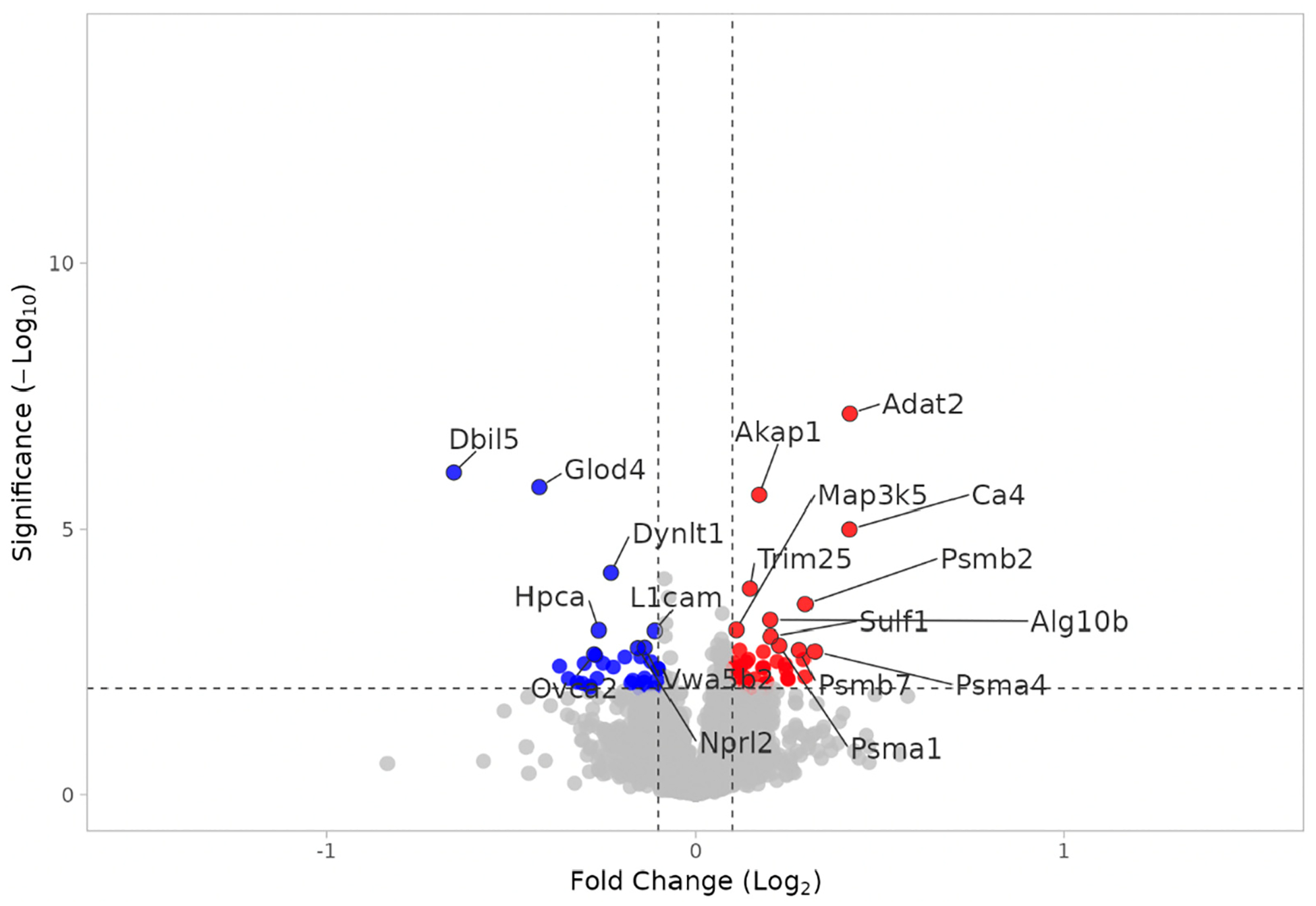
Volcano plot of DEPs in KO vs. WT. MEST/SHAM combined. Upregulated (red) and downregulated (blue) DEPs. X axis: Log2(fold change). Y axis: −Log10(*p* value).

## Data Availability

The original contributions presented in this study are included in the article. Further inquiries can be directed to the corresponding author.
